# Supplementary material for: Robotic Stereotactic Body Radiation Therapy for Oligometastatic Liver Metastases: A Systematic Review of the Literature and Evidence Quality Assessment
Source: Diagnostics (Basel). 2024 May 19;14(10):1055. doi: 10.3390/diagnostics14101055 (PMC11487420; doi:10.3390/diagnostics14101055)
Supplement: Supplementary file 1 [file diagnostics-14-01055-s001.zip › Supplem Table S2.pdf]

Supplementary Table S2: Response rates of included patients.

		Response rate (lesions, %)			
**Authors**	**Total lesions**	**Complete response %**	**Partial response**	**Stable disease**	**Progressive disease**
Vernaleone et al. (11)	63	5 (7.9%)	24 (38.1%)	16 (25.4%)	18 (28.6%)
Anstadt et al. (12)	81	45 (55.6%)	17 (21%)	7 (8.6%)	12 (14.8%)
Stintzing et al. (13)	194	107 (65.2%)	22 (13.4%)	27 (16.5%)	9 (5.5%)
Dutta et al. (14)	33	0	3 (17%)	7 (19%)	8 (44.8%)
Berkovic et al. (15)	55	22 (40.7%)	8 (14.8%)	8 (14.8%)	10 (18.5%)
Garcia et al. (16)	17	4 (23.5%)	3 (17.6%)	3 (17.6%)	1 (5.9%)
Vautravers-Dewas et al. (22)	62	41 (38.9%)	8 (13.1%)	5 (8%)	6 (10%)
Stintzing et al. (23)	54	14 (38.9%)	11 (30.5%)	6 (16.7%)	5 (13.9%)
Stintzing et al. (24)	19	0	9 (64.3%)	5 (35.7%)	5 (35.7%)
Ambrosino et al. (25)	27	7 (25.9%)	13 (48.1%)	3 (11.1%)	4 (14.8%)
**Total**	**605**	**245 (40.5%, 95% CI, 36.66-44.46%)**	**115 (19.01%, 95% CI, 16.07-22.33%)**	**87 (14.38%, 95% CI, 11.8-17.41%)**	**80 (13.22%, 95% CI, 10.74-16.17%)**
