# Supplementary material for: Robotic Stereotactic Body Radiation Therapy for Oligometastatic Liver Metastases: A Systematic Review of the Literature and Evidence Quality Assessment
Source: Diagnostics (Basel). 2024 May 19;14(10):1055. doi: 10.3390/diagnostics14101055 (PMC11487420; doi:10.3390/diagnostics14101055)
Supplement: Supplementary file 1 [file diagnostics-14-01055-s001.zip › Supplemental Table S1.pdf]

Supplemental Table S1. Quality assessment of included case series using the NHLBI Quality Assessment Scale for case series

First Author	Was the study objective clearly stated?	Was the study population clearly and fully described, including a case definition?	Were the cases consecutive?	Were the subjects comparable?	Was the intervention clearly described?	Were the outcome measures clearly defined, valid, reliable, and implemented consistently across all study participants?	Was the length of follow-up adequate?	Was the methodology well-described?	Were the results well-described?	Total score	Quality rating
**Vermaleone et al. [11]**	1	1	1	0	1	1	1	1	1	8	High
**Andratschke et al. [17]**	1	1	1	0	1	1	0	1	1	7	High
**Stintzing et al. [13]**	1	1	1	0	1	1	1	1	0	7	High
**Vautravers-Dewas et al. [22]**	1	1	1	0	1	1	1	1	1	8	High
**Janoray et al. [18]**	1	0	1	0	1	1	1	1	0	6	Fair
**Stintzing et al. [13]**	1	1	1	0	1	1	1	1	1	8	High
**Stintzing et al. [23]**	1	1	1	0	1	1	1	1	1	8	High
**Dewas et al. [21]**	1	1	1	0	1	1	1	1	0	7	High
**Stintzing et al. [24]**	1	1	1	0	1	1	1	1	0	7	High
**Ambrosino et al. [25]**	1	1	1	0	1	1	1	1	1	8	Fair
**Anstadt et al. [12]**	1	1	1	0	1	1	1	1	1	8	Fair
**Berkovic et al. [15]**	1	1	1	0	1	1	1	1	1	8	Fair
**Garcia et al. [16]**	1	1	1	0	1	1	1	1	1	8	Fair
**Dutta et al. [14]**	1	1	1	0	1	1	1	1	1	8	Fair
**Yuan et al. [19]**	1	1	1	0	1	1	1	1	1	8	Fair

The NHLBI scale ranges from 1 – 9; with a score of 1–3 denoting poor quality, 4-6 fair quality, and 7-9 suggesting high quality.
NHLBI: National Heart, Lung, and Blood Institute
